# Mature Myotubes Generated From Human-Induced Pluripotent Stem Cells Without Forced Gene Expression

**DOI:** 10.3389/fcell.2022.886879

**Published:** 2022-05-30

**Authors:** Kei Fujiwara, Risa Yamamoto, Tomoya Kubota, Atsutoshi Tazumi, Tomoka Sabuta, Masanori P. Takahashi, Hidetoshi Sakurai

**Affiliations:** ^1^ Center for iPS Cell Research and Application (CiRA), Kyoto University, Kyoto, Japan; ^2^ Clinical Neurophysiology, Department of Clinical Laboratory and Biomedical Sciences, Division of Health Sciences, Osaka University Graduate School of Medicine, Osaka, Japan; ^3^ Laboratory for Pharmacology, Pharmaceutical Research Center, Asahi Kasei Pharma Corporation, Shizuoka, Japan

**Keywords:** myogenic differentiation, mature myotube, human iPS cell, transgene free, screening tools

## Abstract

Human-induced pluripotent stem cells (hiPSCs) are a promising tool for disease modeling and drug screening. To apply them to skeletal muscle disorders, it is necessary to establish mature myotubes because the onset of many skeletal muscle disorders is after birth. However, to make mature myotubes, the forced expression of specific genes should be avoided, as otherwise dysregulation of the intracellular networks may occur. Here, we achieved this goal by purifying hiPSC-derived muscle stem cells (iMuSC) by Pax7-fluorescence monitoring and antibody sorting. The resulting myotubes displayed spontaneous self-contraction, aligned sarcomeres, and a triad structure. Notably, the phenotype of sodium channels was changed to the mature type in the course of the differentiation, and a characteristic current pattern was observed. Moreover, the protocol resulted in highly efficient differentiation and high homogeneity and is applicable to drug screening.

## Introduction

Many congenital and late-onset myopathies are characterized by the malformation of intracellular structures, such as the triad structure, which is composed of t-tubules and the sarcoplasmic reticulum (SR) (Buj-Bello et al., 2008; Al-Qusairi et al., 2009; Cowling et al., 2014), sarcomeric structures (Li et al., 2020), and nuclei morphology (Davidson and Lammerding, 2014). To investigate the pathophysiology of these disorders *in vitro*, it is necessary to generate mature myotubes with intramuscular structures that form in the late-stage development.

Human-induced pluripotent stem cells (hiPSCs) are a powerful tool for analyzing the pathophysiology of various disorders. In the field of skeletal muscle biology, efficient and stable differentiation methods for the generation of myotubes from hiPSCs have been reported. We generated myotubes by driving the transcription factor *MyoD*, a myogenic gene, exogenously (Tanaka et al., 2013). In this method, myotubes are differentiated directly from hiPSCs for 6–7 days with high purity and efficiency. Using this method, we have successfully established disease models of muscular diseases such as dysferlinopathy (Tanaka et al., 2013), Duchenne muscular dystrophy (Shoji et al., 2015), Pompe disease (Yoshida et al., 2017), and myotonic dystrophy (Ueki et al., 2017). Following the model for dysferlinopathy, a drug screening system was established (Uchimura et al., 2017), and an effective chemical for treatment (Nocodazole) was found (Kokubu et al., 2019). However, these *MyoD*-mediated myotubes are immature and show neither the sarcomeric structure nor the triad structure, making them unsuitable for studying skeletal muscle diseases that occur with late-stage development. Another transcription factor commonly expressed exogenously is *Pax7* (Darabi et al., 2012). Using muscle stem cells obtained by lentiviral-expressed *Pax7*, several groups have produced mature myotubes (Rao et al., 2018; Selvaraj et al., 2019). However, the exogenous expression may disrupt intracellular gene networks.

Skeletal muscles are huge organs with electrical excitability, which is precisely organized by several ion channels and transporters. Among them, the voltage-gated sodium channel (Nav) plays an essential role as the generator of action potentials (Ahern et al., 2016). In the mature skeletal muscle, Nav1.4, which is encoded by *SCN4A* gene, is expressed dominantly, whereas the immature skeletal myotube is known to express a significant amount of Nav1.5, which is encoded by *SCN5A* gene, which is primarily expressed in cardiac myotubes (Yang et al., 1991) (Martínez-Mármol et al., 2007). A well-known difference between them is sensitivity for tetrodotoxin (TTX); Nav1.4 is a TTX-sensitive Nav channel (IC_50_ = 25 nM), whereas Nav1.5 is TTX-resistant (IC_50_ > 1 μM) (Chanine et al., 1994). In addition, electrophysiological experiments using the heterologous expression system have shown that the voltage dependence of Nav1.5 is shifted in the hyperpolarized direction compared to that of Nav1.4; Nav1.5 activates at a lower voltage than Nav1.4 (Sheets and Hanck, 1999; Vilin et al., 2012). These properties influence the physiological excitability of skeletal muscles so that electrophysiological assessments are also important to evaluate the maturity of myotubes from hiPSCs.

Recently, we established a protocol to prepare hiPSC-derived muscle stem cells (iMuSCs) using small molecules without driving transcription factors exogenously (Zhao et al., 2020). Using these iMuSCs, here we developed a novel method to produce mature myotubes with well-aligned sarcomeric structures and triad structures *in vitro*. These myotubes displayed mature skeletal muscle characteristics not only in gene expression patterns but also in electrical excitability. Additionally, we differentiated iMuSCs to myotubes with high efficiency and homogeneity in 96 multi-well plates. These findings enable disease modeling and drug screening of congenital and late-onset myopathies using disease-specific hiPSCs.

## Results

### Preparation of hiPSC-Derived Muscle Stem Cells (iMuSCs) by Fluorescence Monitoring and Antibody Selection

An illustration of our previous method to generate fetal iMuSCs is shown in Supplementary Figure S1A (Zhao et al., 2020). To isolate these iMuSCs, hiPSCs carrying a *PAX7* (satellite cell marker)-Venus reporter was used (Nalbandian et al., 2021). After approximately 80 days of culture, Venus-positive cells appeared in the differentiation culture (Supplementary Figure S1B) and were purified by flow cytometry (Supplementary Figure S1C). To replace the *PAX7*-Venus reporter hiPSCs, we developed a purification method using antibodies for surface markers. Although several surface markers are reported, we utilized CD82, a marker of progenitor cells in human skeletal muscles (Alexander et al., 2016; Uezumi, A. et al., 2016), as a positive selection marker, and CD57 (also known as HNK-1), a marker of neural crest cells, as a negative selection marker for iMuSCs (Borchin et al., 2013). After staining day-80 differentiated cells with CD82 and CD57 antibodies (see *Methods*), the differentiated cells were separated into four fractions: CD57^+^CD82^+^, CD57^−^–CD82^+^, CD57^+^CD82^−^, and CD57^−^–CD82^−^ (Supplementary Figure S2A). A gene expression analysis revealed that the CD57^−^–CD82^+^ fraction showed the highest expression level for several muscle stem/progenitor markers including *PAX7, MYF5,* and *MYOD* among the four fractions (Supplementary Figure S2B). An immunocytochemical analysis demonstrated that more than 80% of CD57^−^–CD82^+^ cells expressed *PAX7* (Supplementary Figure S2C). Together, these results suggest that cell sorting the CD57^−^–CD82^+^ fraction efficiently purifies iMuSCs from non-reporter hiPSCs. Thus, we could obtain iMuSCs by *PAX7*-Venus fluorescence monitoring and by CD57^−^–CD82^+^ antibody selection.

### Optimization of Myotube Differentiation From Purified iMuSCs

**FIGURE 1:**
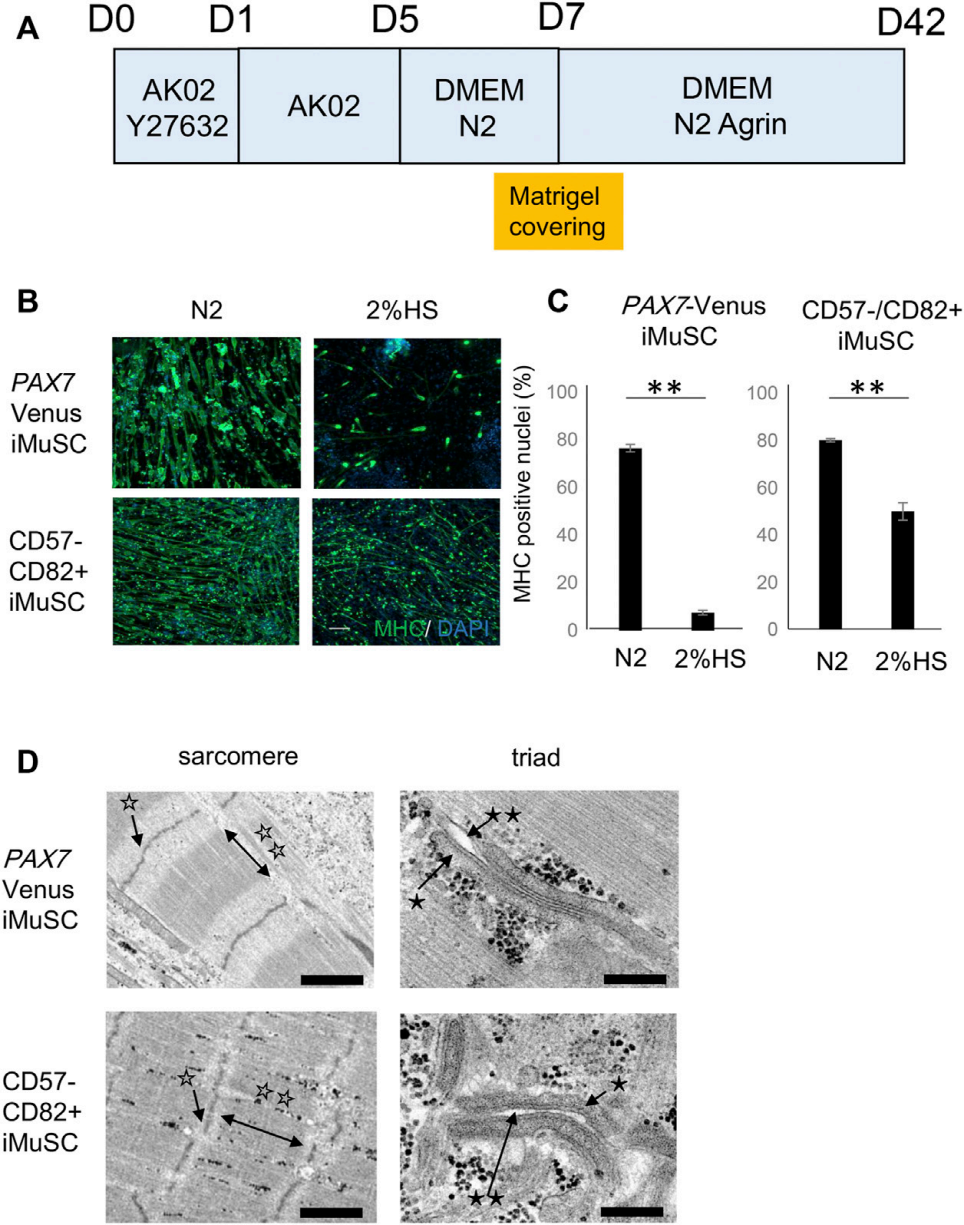
Generation of mature myotubes from iMuSCs *in vitro*. **(A)** Schematic of the myotube differentiation schedule. The proliferation phase of iMuSCs is from days **(D)** 0 to 5. D5 is when the medium is switched to the differentiation medium. D7 is when the 3D Matrigel covering is done. **(B)** Representative images of myotubes (anti-MHC green) in 2% HS and N2 supplement at D14. Scale bar, 1,000 µm. **(C)** Ratio of nuclei of MyHC-positive myotubes to total nuclei in 2% HS and N2 supplement at D14. The ratio was higher in the N2 medium compared with that in the HS medium for both Pax7-Venus iMuSC-derived myotubes (left) and CD57^−^–CD82^+^ iMuSC-derived myotubes (right). Data are represented as the mean ± SEM. **p < 0.01, unpaired two-tailed Student’s t-test. **(D)** Representative electron microscopic images of myotubes in m-condition (Matrigel embedding) at D42. Well-aligned sarcomeric structures and triad structures are seen in Pax7-Venus iMuSC-derived myotubes (upper panel) and CD57^−^–CD82^+^ iMuSC-derived myotubes (lower panel). The white single star (☆) indicates the Z-line, white double stars (☆☆) indicate the A-line, the black single star (*) indicates the sarcoplasmic reticulum, and black double stars (**) indicate t-tubules. Scale bars, 250 nm (PAX7-Venus sarcomere), 200 nm (PAX7-Venus triad), 1,000 nm (CD57^−^–CD82 ^+^ sarcomere), and 200 nm (CD57^−^–CD82 ^+^ triad).

We then optimized the *in vitro* myotube differentiation of the purified iMuSCs. Figure 1A shows a simple scheme of the *in vitro* myotube differentiation protocol. iMuSCs were proliferated to 100% confluence by AK02 media for 5 days (Supplementary Figure S3A), at which time we examined the component of differentiation media that affected the myotube differentiation efficiency. N2 supplement was found to promote the induction of myosin heavy chain (MHC)-positive myotubes, which is a hallmark of myogenic differentiation efficiency compared with 2% horse serum (HS) supplement (Figures 1B,C).

### Generation of Mature Myotubes With Sarcomere and Triad Structures

After switching to differentiation media with N2 supplement, iMuSCs displayed a spindle-like morphology at day 7 of the culture (Supplementary Figure S3B). Generally, myotubes are surrounded by connective tissues, such as extracellular matrix, *in vivo*. To imitate this environment, we developed a novel method to embed a high concentration of Matrigel to the culture dish *in vitro* (Supplementary Figure S4A) by referring to a previous report (Falcone, S. et al., 2014) that demonstrated murine primary satellite cell maturation *in vitro*. After embedding the Matrigel, iMuSC-derived myotubes began to fuse to each other and displayed spontaneous self-contractions at day 14 of the culture (Supplementary Figure S3C and Supplementary Movie S1). This feature was more obvious at day 42 of the culture (Supplementary Figure S3D).

Mature skeletal muscle is equipped with structures for contraction, such as the sarcomere and triad structures, which is composed of t-tubules and the SR. At day 42 of the culture, the intramuscular structure was evaluated by electron microscopy (Figure 1D). Well-aligned sarcomere and triad structures were visible, suggesting that the myotubes were considerably mature.

Finally, we evaluated the effect of Matrigel embedding by comparing the condition with Matrigel embedding (m-condition) to without Matrigel embedding (c-condition) (Supplementary Figure S4B). The size of the myotubes was measured between two conditions at day 42 of the culture to assess the maturation level of myotubes. m-condition increased the myotube diameter by 16.5% (*p* < 0.013) if using *PAX7*-Venus iMuSCs and by 19.8% if using CD57^−^–CD82^+^ iMuSCs (*p* < 0.009) (Supplementary Figures S4C,D).

### Expression of Genes Involved in *MYH* Subtypes and Calcium Homeostasis is Characteristic of Myotube Maturation

**FIGURE 2:**
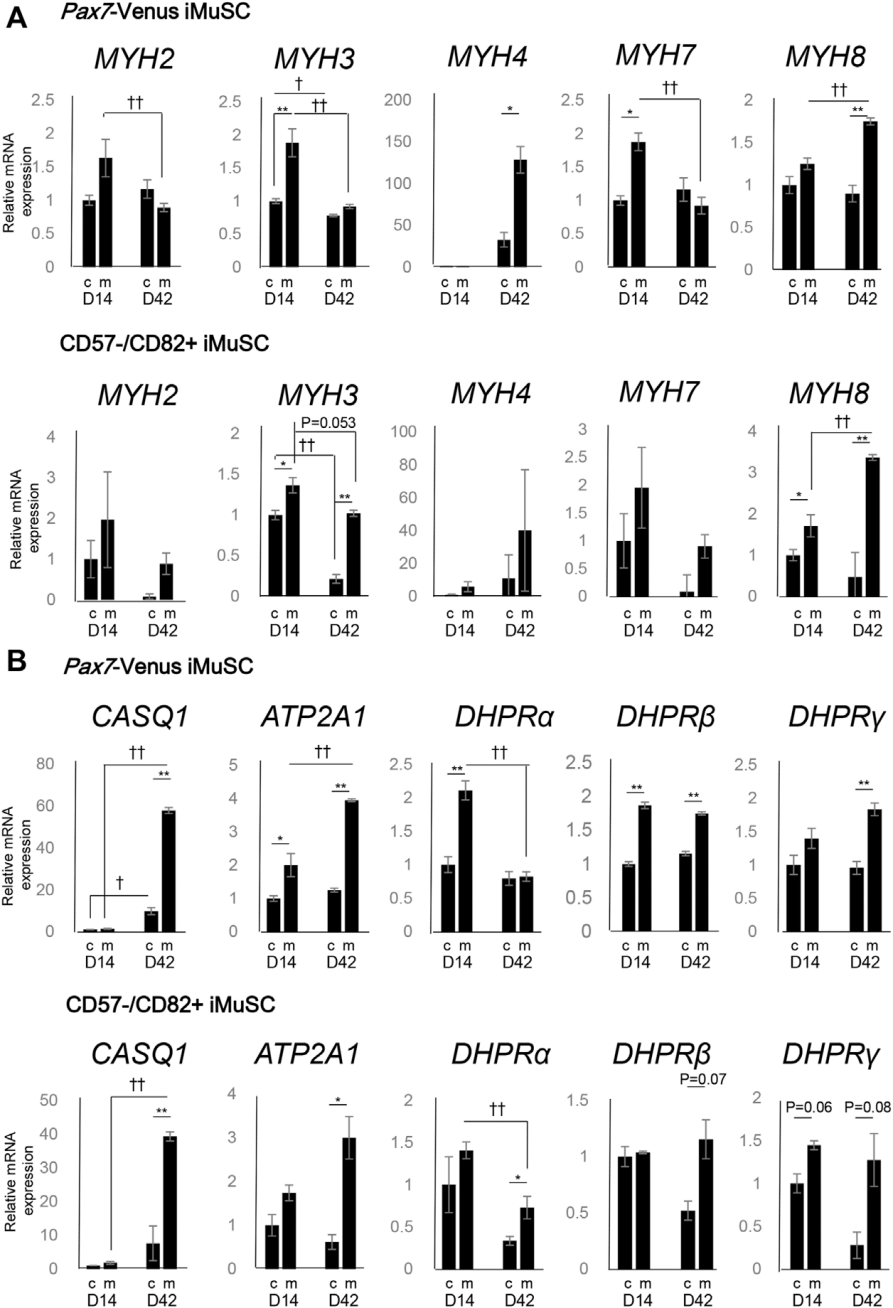
Myosin heavy chain (MYH) subtypes and triad-associated genes display characteristic expression patterns. **(A)** The mRNA expression of the myosin heavy chain family (MYH2, MYH3, MYH4, MYH7, and MYH8) was quantified by real-time PCR. The expression levels of the MYH genes in PAX7-Venus iMuSC-derived myotubes (upper) and in CD57^−^–CD82^+^ iMuSC-derived myotubes (lower) on days 14 and 42 in c-condition (no Matrigel embedding) and m-condition (Matrigel embedding) are shown. **(B)** The mRNA expression of SR-related genes (Casq1 and Atp2a1) and t-tubule-related genes (DHPRα, DHPRβ, and DHPRγ) was quantified by real-time PCR. The expression levels of MYH genes in PAX7-Venus iMuSC-derived myotubes (upper) and in CD57^−^–CD82^+^ iMuSC-derived myotubes (lower) on days 14 and 42 in c-condition and m-condition are shown. The vertical axis of the graph shows the relative expression level normalized by 18S rRNA, the housekeeping gene. The expression level is standardized by the expression level (=1) on day 5. *p < 0.05, **p < 0.01; c-condition vs. m-condition. †*p* < 0.05, ††*p* < 0.01; day 14 vs. day 42 (unpaired two-tailed Student’s t-test). Error bars indicate the standard error of mean (SEM). In each group (day 14c, day 14m, day 42c, and day 42m), three independent samples were used for the analysis.

To assess the maturity of differentiated myotubes, the expression of *MYH* genes was investigated between c-condition and m-condition at days 14 and 42 using *PAX7*-Venus and CD57^−^–CD82^+^ iMuSC-derived myotubes. The expression of *MYH3* (embryonic subtype) was decreased from day 14 to day 42 in both iMuSC-derived myotube types (Figure 2A). This pattern of expression, which indicates a shift from developmental to adult myosin, is consistent with the findings during native muscle development (Schiaffino et al., 2015) and differentiation (Brown et al., 2012). Moreover, the expression of *MYH3* itself was higher in m-condition than in c-condition.

On day 14, the expression of four different *MYH* subtypes associated with postnatal to adult development (*MYH2,4,7,8*) was elevated. Especially on day 42, the expression of *MYH4* was elevated in m-condition compared with that in c-condition in *PAX7*-Venus iMuSC-derived myotubes (Figure 2A).

Moreover, the expression of genes related to SR and t-tubules was investigated. On day 42, the expression of SR-related markers (*CASQ1* and *ATP2A1*) was notably elevated in m-condition compared with that in c-condition in *PAX7*-Venus and CD57^−^–CD82^+^ iMuSC-derived myotubes (Figure 2B). Also on day 42, the expression of t-tubule-related genes (*DHPRβ* and *DHPRγ*) was elevated in m-condition in both myotube types (Figure 2B). Altogether, these results suggest that m-condition promotes mature myotubes *in vitro* more than c-condition.

Finally, the expression of *MYH*, t-tubules, and SR related genes was investigated in m-condition with or without Agrin to clarify the effect of Agrin for myotube differentiation. The expression of most genes was comparable between with and without Agrin except a slight difference of *MYH3* and *DHPRβ* at day 42. These data indicate that Agrin itself does not have significant effect on the expression of myogenic markers (Supplementary Figure S5). However, the triad structure was observed only in the presence of Agrin (Figure 1D) and never detected without Agrin condition (data not shown), suggesting that Agrin might have an important role for forming the triad structure.

### Myotubes Display Characteristic Electric Properties Related to Sodium Channels

**FIGURE 3:**
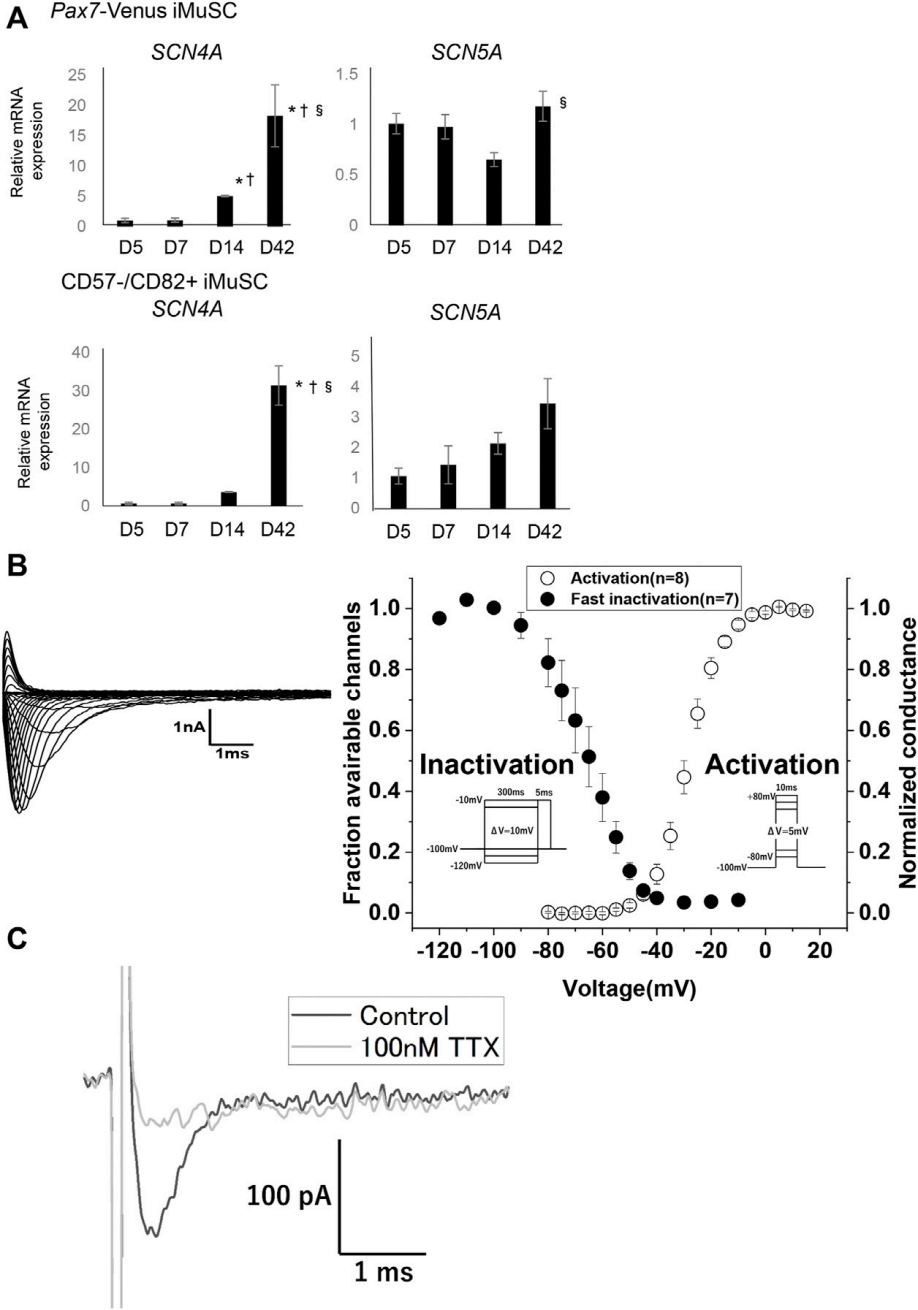
Myotubes have voltage-dependent Na⁺channels, most likely Nav1.4. **(A)** The mRNA expressions of SCN4A, which codes Nav1.4, and SCN5A, which codes Nav1.5, were quantified by real-time PCR. The expression levels of SCN4A and SCN5A in PAX7-Venus iMuSC- and CD57^−^–CD82^+^ iMuSC-derived myotubes on days 5, 7, 14, and 42 are shown. The vertical axis shows the relative expression level normalized by 18S rRNA, the housekeeping gene. The expression of SCN4A gradually elevated with time. Error bars indicate the standard error of mean (SEM). In each group (days 5, 7, 14, 42), three independent samples were used for the analysis. *p < 0.05; vs. day 5. †*p* < 0.05; vs. day 7. §*p* < 0.05; vs. day 14. **(B)** Representative current traces elicited by the activation protocol in PAX7-Venus iMuSCs around day 7 of the culture (left). Voltage dependence of activation (right, open circles) and steady-state fast inactivation (left, filled circles) (right). **(C)** Representative current obtained by the out-side-out patch clamp technique. The black trace indicated the Na + current without TTX elicited by -10 mV test pulse from −100 mV of holding potential. The gray trace indicated the Na + current from the identical cell in the presence of 100 nM TTX elicited by the same test pulse.

Next, to assess physical maturity, the electrical properties of the myotubes derived from iMuSC were investigated. The gene expressions of *SCN4A* and *SCN5A* were investigated in both *PAX7*-Venus and CD57^−^–CD82^+^ iMuSC-derived myotubes (Figure 3A). Interestingly, *SCN4A* expression both in *PAX*7-Venus and in CD57^−^–CD82^+^ iMuSC-derived myotubes was increased dramatically in a time-dependent manner with the culture. The expression of *SCN5A* was increased in CD57^−^–CD82^+^ iMuSC-derived myotubes as the cells matured but was relatively stable in *PAX7*-Venus iMuSC-derived myotubes. These results suggest that Nav1.4 is dominant in both *PAX7*-Venus and CD57^−^–CD82^+^ iMuSC-derived myotubes.

Additionally, voltage-dependent sodium currents were measured in *PAX7*-Venus iMuSC-derived myotubes using the whole-cell patch-clamp technique. Representative raw traces of sodium currents recorded from cultured cells around day 7 of the culture are shown (Figure 3B left). Transient currents were elicited in a voltage-dependent manner, suggesting that currents were derived from Nav channels. To characterize the sodium currents, the kinetics of the channel gating was examined. The voltage dependence of the activation (Figure 3B, right, open circles) and the steady-state fast inactivation (Figure 3B, filled circles) are shown. The estimated parameters for the intermediate potentials $\left(V_{1/2}\right)$ and slope factors (k) are listed in Supplementary Table S2. Next, we investigated the TTX sensitivity of the Na^+^ current by the out-side-out patch clamp technique, which enables us to expose the membrane to TTX without physical prevention by the Matrigel. In the membrane excised from the myotube around day 11, approximately 140 pA of Na^+^ current was recorded in the conventional bath solution without TTX (Figure 3C, black). After exposure of 100 nM TTX, the current was blocked nearly completely (Figure 3C, gray), suggesting that the excitability of *PAX7*-Venus iMuSC-derived myotubes relies on the expression of Nav1.4 rather than Nav1.5.

### iMuSCs can be Utilized for Multi-Well Screening

**FIGURE 4:**
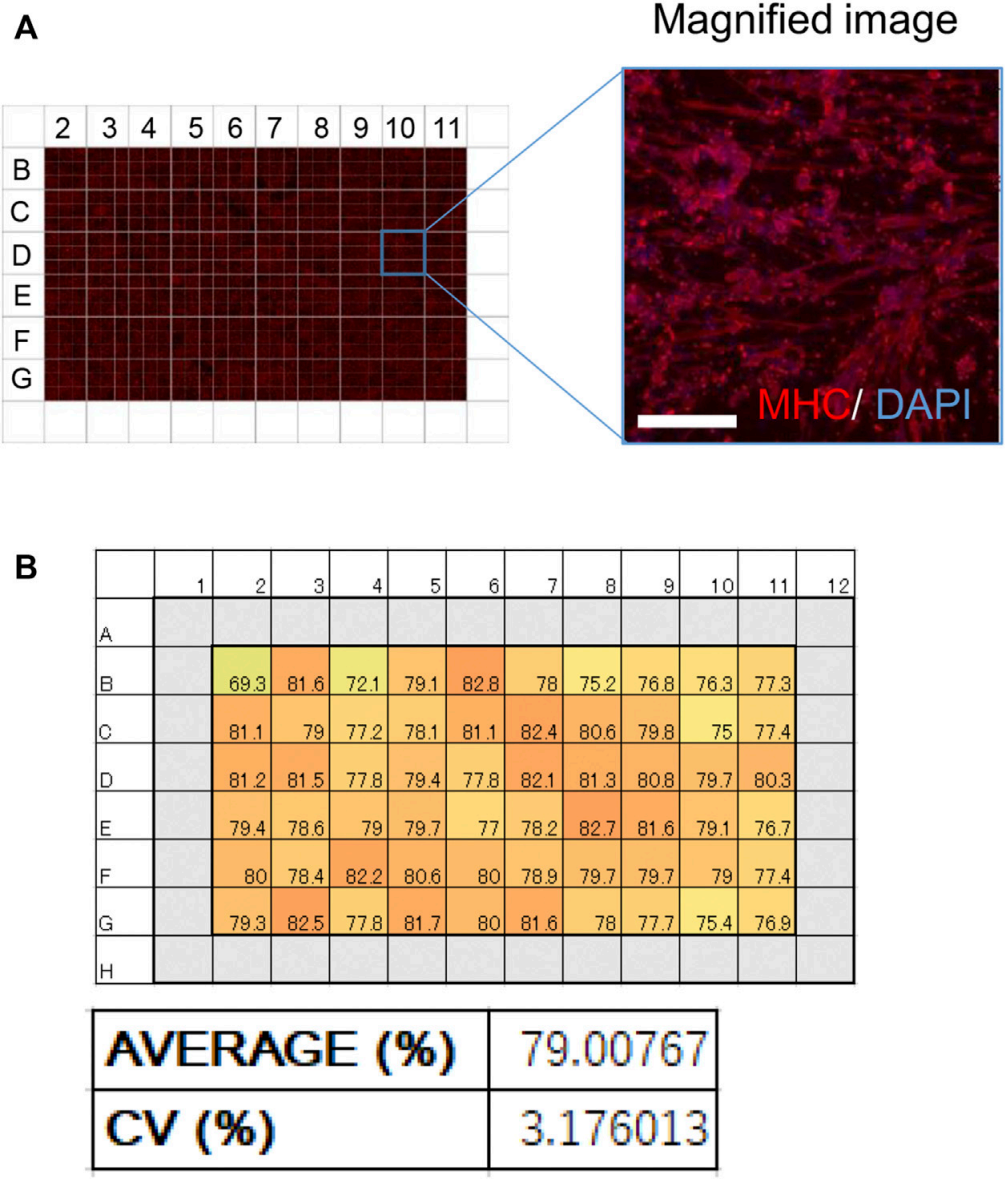
Effective and homogeneous differentiation system in a 96-well plate. **(A)** An image of MHC-stained myotubes (red) in a 96-well plate. A magnified image is also shown. Scale bar, 100 μm. **(B)** Schematic of the 96-well plate used in the screening. (Upper) Numbers in every well indicate the ratio of MHC-positive nuclei to total nuclei (%). (Lower) A bar graph of the ratio of MHC-positive nuclei in the 96-well plate. The average ratio of MHC-positive nuclei and the coefficient of variation (CV) are shown.

hiPSC-based phenotype screening can be used to develop novel drugs (Yamashita, A et al., 2014). To use our myotubes as a resource for drug discovery, iMuSCs were differentiated to myotubes in a 96-well plate to model a multi-well drug screening system. Only the internal 60 wells in the plate were used because external margins of the plate are not always suitable due to instability of the cell culture (Lundholt, BK et al., 2003). At 28 days of culture, iMuSCs in each of the 60 wells were differentiated to myotubes with high homogeneity and positive for MHC (Figure 4A). The average differentiation efficiency was over 79%, and the coefficient of variation number (CV number) was under 3.3% (Figure 4B), suggesting that this model satisfied criteria for a multi-well drug screening system.

## Discussion

In this article, we succeeded in generating mature myotubes in a transgene-free condition with high efficiency using hiPSC-derived iMuSCs. Because this method does not require unique devices, it is relatively easy to reproduce myotube differentiation. Mature myotubes were generated using commercially available items such as Matrigel, growth factors, and 96-well cell culture plates. Therefore, this multi-well differentiation system is applicable to disease modeling and drug screening.

In the field of skeletal muscle biology, hiPSCs are a promising tool for disease modeling and phenotypic screening (Ortiz-Vitali and Darabi, 2019). However, at present, it is difficult to make mature, physiological myotubes from hiPSCs that are reliable for phenotypic screening. Although our colleague demonstrated that screening applicable mature myocytes could be induced by the MyoD-mediated differentiation method, a contractile stimulation by electric field stimulation is still necessary to induce the sarcomeric structure (Uchimura et al., 2021). Therefore, we aim to develop a more simple method to generate screening applicable mature myotubes from hiPSCs in this study.

Recently, some groups succeeded in making mature myotubes from hiPSCs characterized by the formation of the triad structure by exogenously driving the *PAX7* gene (Rao et al., 2018; Selvaraj et al., 2019). However, this approach may result in abnormal transcriptional networks, which could have adverse effects on the pathophysiological analysis of various muscular diseases. Myotubes generated using growth factors or nutrient factors without exogenous gene expression could solve this problem (Caron et al., 2016; Swartz et al., 2016). However, myotubes generated by chemical compounds lack properties characteristic of mature myotubes, such as the triad structure and elevated *SCN4A* gene expression. Another way to generate mature myotubes is to use specific devices. For example, silicone posts providing continuous tension promote the maturation of hiPSC-derived myotubes (Mafioletti et al., 2018). However, such devices are not suitable for phenotype screening because their material is not easily transferable to such systems. On the other hand, the present study realized mature myotubes that are applicable to phenotype screening.

Several reports have suggested that myotube culture with hydrogel covering promotes differentiation and maturation (Maffioletti et al., 2018; Jensen et al., 2020; Urciuolo et al., 2020). In these reports, Matrigel diluted in the differentiation medium was used as the hydrogel material, and the myotubes indicated maturation by the expression change of various *MYH* gene subtypes. In an environment of Matrigel embedding, myotubes are fully covered with extracellular matrix from all directions, mimicking the environment observed *in vivo*. This condition is likely more suitable for the differentiation of mature myotubes because laminin, entactin, and collagen IV, which are all major components of Matrigel, play important roles in muscle differentiation and maturation (Lee et al., 2015; Penton et al., 2016).

In mature skeletal muscle, Nav1.4 is dominantly expressed, whereas the expression of Nav1.5 is predominant in immature and denervated skeletal muscle (Kallen et al., 1990) (Yang et al., 1991). A previous report showed that Nav1.4 activates at the more depolarized voltage compared with Nav1.5 (Sheets and Hanck, 1999) (Vilin et al., 2012). In addition, the expression ratio of Nav1.4 and Nav1.5 can influence the excitability of the skeletal muscle, such as its sensitivity to pH (Vilin et al., 2012) and intracellular calcium (Ben-Johny et al., 2014). Transient Na^+^ currents recorded from *Pax7*-Venus iMuSC-derived myotubes in this study showed that the voltage dependence of activation was more depolarized than that from hiPSC-derived cardiac myotubes (Selga et al., 2018). Moreover, our out-side-out patch clamp recording showed that the Na^+^ currents are TTX-sensitive, indicating the existence of a large amount of Nav1.4. These results supported that *Pax7*-Venus iMuSC-derived myotubes implement physiological excitability comparable to the normal skeletal muscle.

A major advancement of hiPSC applications is the detection of therapeutic chemicals by large-scale screening (Ortiz-Vitali and Darabi, 2019). To apply our method accordingly, it is necessary to establish a multi-well system with homogeneous differentiation capacity. CV is an index that measures the variation in each well, and previous reports suggest that a CV<15% is desirable for drug screening (Lariosa-Willingham, K. D. et al., 2016), which is a criterion that our system achieved.

To conclude, we generated mature myotubes without driving exogenous genes. The myotubes displayed a mature character with regard to morphology, gene expression patterns, and sodium channel properties. Thus, our differentiation method for myotubes may be a novel approach to study disease pathophysiologies and to identify therapeutic chemicals for skeletal muscle disorders.

## Experimental Model and Subject Details

### Human iPSC Lines and Maintenance

Experiments using hiPSCs were approved by the Ethics Committee at the Graduate School and Faculty of Medicine Kyoto University (approval numbers #E1762, #G567, and #Rinsho71). This study was performed conforming to the guidelines of the Declaration of Helsinki and was conducted after obtaining written informed consent.

The hiPSC line 201B7 was generated from purchased fibroblasts of a healthy donor by retroviral transduction (Takahashi et al., 2007). Ff-WJ14s516 (an HLA-homozygous hiPSC line with the most frequent haplotype in Japan; abbreviated as S516 in this manuscript) was established from cord blood cells by an episomal vector system (Okita et al., 2013) at the Facility for iPS cell Therapy, CiRA, Kyoto University. HiPSCs were cultured in StemFit media (AK02N; Ajinomoto) and passaged once a week. At the passage, the cells were completely detached from a 3.5-cm well in a dish by incubating in 500 μl of Accutase (Nacalai Tesque) for 10 min followed by neutralization with 2 ml of Stemfit media. After pipetting gently, 3 × 10⁵ hiPSCs were seeded on the dish in Stemfit media containing a Rock inhibitor, 10 μM Y-27632 (Nacalai Tesque). The medium was replaced with 1.5 ml of StemFit media without the Rock inhibitor 2, 4, 5, and 6 days after passage. The maximum passage number was 30.

## Method Details

### Purification of iMuSCs

The myogenic differentiation of hiPSCs was performed following a previous protocol (Zhao M. et al., 2020). Cell populations including fetal iMuSCs were obtained at days 80–100. The purification of iMuSCs was performed by FACS sorting (BD FACS aria 650110J1). For the 201B7-*Pax7* Venus line (Nalbandian et al., 2021), the iMuSCs were purified by Venus fluorescence. For non-reporter lines, iMuSCs were purified as the CD57^−^–CD82^+^ population. Allophycocyanin-conjugated CD82 antibody (Biolegend 342108) was used at a final concentration of 1:100. Phycoerythrin-conjugated CD57 antibody (BD Pharmingen 560844) was used at a final concentration of 1:200. The cells were stained with the antibodies for 30 min, followed by washing with HBSS buffer once.

### Expansion and Differentiation of iMuSCs

Purified iMuSCs were plated onto a 1:100 Matrigel growth factor reduced (Corning 354230) in a StemFit media-coated 96-well microplate (IWAKI 3860–096). The microplate was pre-coated with Matrigel diluted by StemFit media at 1:100 for 1 h at 37°C. Then, 5,000 purified iMuSCs were seeded onto the plate and cultured in StemFit media containing 10 μM Y-27632 for expansion.

iMuSCs started to proliferate and reach confluency after approximately 5 days of culture. In this process, the medium was changed every day until day 5. After the day of seeding, the medium was replaced to Stemfit media without Y-27632. Differentiation was induced by switching to differentiation media composed of DMEM (Invitrogen 11885084) supplemented with 1% N2 supplement (Gibco 17502048) or 2% HS (Sigma H1138). Recombinant rat Agrin (R&D Systems 550-AG) was used for better differentiation.

### Matrigel Covering and Agrin Addition

At day 7 of the culture, 3D Matrigel covering was performed. A Matrigel mixture composed of cold DMEM media (40 μl) and Matrigel (20 μl) was prepared. First, the differentiation media was completely removed from the well, and myotubes were covered with the Matrigel mixture as previously described (Falcone S. et al., 2014). Afterward, the culture dish was incubated at 37°C for 30 min. As a result, myotubes were covered with a high concentration of Matrigel mixture. Next, the differentiation medium containing Agrin at a final concentration of 80 μg/ml was gently added on the 3D Matrigel. Finally, the culture dish was moved to a 37°C incubator, and the medium was changed every 2 days. After the Matrigel coating, Agrin-containing DMEM/N2 media was used as the differentiation media.

### RNA Isolation and Quantitative Polymerase Chain Reaction (qPCR)

Total RNA was isolated using a ReliaPrep RNA cell Miniprep Kit (Promega Z6012) according to the manufacturer’s instructions. Reverse transcription was performed by ReveTra Ace qPCR RT Mater Mix with gDNA remover (TOYOBO FSQ-301). SYBR Green reagent (Applied Biosystems) was used to detect the target sequences. qPCR was performed using StepOnePlus. (Applied Biosystems) The list of primers used in this study is shown in Supplementary Table S1.

### Immunostaining

Myotubes were fixed with PBS containing 2% (w/v) paraformaldehyde for 10 min at 4°C. Samples were washed with PBS twice, followed by blocking with Blocking One (Nacalai Tesque) for 60 min at 4 °C and incubated overnight at 4 °C with primary antibodies diluted in 10% (v/v) Blocking One in PBS-T (PBS with 0.2% (v/v) Triton X-100 solution (Nacalai Tesque). The samples were then washed 3 times with PBS-T and incubated for 1 h at room temperature with secondary antibodies diluted in 10% (v/v) Blocking One in PBS-T. Finally, the samples were incubated with DAPI for 5 min to visualize cellular nuclei, followed by washing with PBS twice.

### Analysis of Myosin Heavy Chain-Positive Cells

An analysis of myosin heavy chain (MHC)-positive cells was performed using Arrayscan High-Content Systems (Thermofisher). After immunostaining of the myotubes with MHC antibody, the ratio of nuclei with the surrounding MHC fluorescence to total nuclei was calculated. The threshold was changed depending on the background signal.

### Electron Microscopy

The samples were fixed with 2% paraformaldehyde (PFA) and 2% glutaraldehyde (GA) in 0.1 M phosphate buffer (PB), pH 7.4, at the incubation temperature (37°C) and then put into a refrigerator for 30 min to lower the temperature to 4°C. Thereafter, they were fixed with 2% GA in 0.1 M PB at 4°C overnight. After the fixation, the samples were washed 3 times with 0.1 M PB at 4°C for 1 h. The samples were dehydrated in graded ethanol solutions (50%, 70%, 90%, 100%) as follows: 50% and 70% for 5 min each at 4°C, 90% for 5 min at room temperature, and three changes of 100% for 5 min each at room temperature. Then, the samples were transferred to a resin (Quetol-812; Nissin EM Co., Tokyo, Japan) and polymerized at 60°C for 48 h. The polymerized resins were ultra-thin-sectioned at 70 nm with a diamond knife using an ultramicrotome (Ultracut UCT; Leica, Vienna, Austria) and mounted on copper grids. They were stained with 2% uranyl acetate at room temperature for 15 min and then washed with distilled water, followed by secondary staining with Lead stain solution (Sigma-Aldrich Co., Tokyo, Japan) at room temperature for 3 min. The grids were observed by a transmission electron microscope (JEM-1400Plus; JEOL Ltd., Tokyo, Japan) at an acceleration voltage of 100 kV. Digital images (3,296 × 2,472 pixels) were taken with a CCD camera (EM-14830RUBY2; JEOL Ltd., Tokyo, Japan). Samples were analyzed by Tokai Electron Microscopy, Inc. (Nagoya, Japan).

### Cell Preparation for Current Measurements

To measure sodium currents, a modified culture protocol was used to easily access the patch electrode to an isolated cell. Briefly, 12-mm round glass coverslips (Warner Instruments, Holliston, MA) were used with Matrigel the day before seeding. The cells were harvested on the Matrigel-coated coverslips without overlaying the Matrigel mixture. The myotubes were cultured in the differentiation medium until the patch-clamp experiment.

### Whole-Cell and Out-Side-Out Patch Clamp Recording

Sodium currents were measured using a patch-clamp electrode and the whole-cell recording technique. Recordings were made using an Axopatch 200B amplifier (Molecular Devices, San Jose, CA), and data acquisition was done using a Digidata 1440A (Molecular Devices, San Jose, CA). pCLAMP10.7 software was used (Molecular Devices, San Jose, CA) for the data collection and analysis. Patch electrodes were fabricated from borosilicate glass tubes (Sutter, Novato, CA) using a P-97 Flaming/Brown Micropipette Puller (Sutter, Novato, CA). The electrode’s tip was heat-polished with a final resistance of 1.5–2.3 MΩ in the bath solution. Series resistance was compensated at 80–85% by the analog circuitry of the amplifier. All recordings were conducted at room temperature (25°C). The pipette internal solution contained (in mM) 105 CsF, 35 NaCl, 10 ethylene glycol tetraacetic acid (EGTA), and 10 Cs-HEPES (pH 7.4). The bath solution contained (in mM) 140 NaCl, 4 KCl, 2 CaCl_2_, 1 MgCl_2_, 5 glucose, and 10 Na-HEPES (pH 7.4).

Before starting a measurement, the membrane potential was held at −100 mV for about 5 min to ensure a recovery from slow inactivation. The activation of Na currents was measured by applying a 10 ms depolarizing step pulse from −80 to +80 mV at 5 mV increments. Any cells with peak currents <1 or >10 nA on a step depolarization from −120 mV to −10 mV were excluded. The voltage dependence of the steady-state fast inactivation was measured as peak inward currents elicited by a −10 mV test pulse after a 300 ms conditioning potential of −120 mV to −10 mV. For out-side-out configuration, we prepared electrodes with a final resistance of 1.5 MΩ. A control current without TTX was obtained by a −10 mV test pulse from −120 mV, Then, the current in the presence of 100 nM TTX was obtained by the identical test pulse.

### Validation of Screening System

For the validation of screening system, myotubes differentiated at 28 days with Matrigel covering the *in vitro* culture were used. The myotubes were stained with MHC antibody (Santacruz sc-20641) followed by analysis using a high-content screening instrument (Thermo Scientific Arrayscan System).

## Quantification and Statistical Analysis

### Data Analysis of Electric Currents

Curve fitting was manually performed using Origin (OriginLab Northampton, MA). Conductance was calculated using the following equation:

$$G\left(V\right)=I_{peak}/\left(V-E_{rev}\right)$$

The reversal potential, $E_{rev}$ , was measured experimentally for each cell. The voltage dependence of activation was calculated from the following Boltzmann equation:

$$G\left(V\right)=G_{max}/\left[1+exp\left(-\left(V-V_{1/2}\right)/k\right)\right]$$

Steady-state fast inactivation was fitted to the Boltzmann function calculated from the following equation, where $V_{1/2}$ is the half-maximum voltage, and k is the slope factor:

$$I/I_{max}=1/\left[1+exp\left(\left(V-V_{1/2}\right)/k\right)\right]$$

$I_{max}$ was calculated as the average of the three I values before and after the potential, where I shows the maximum point.

### Statistical Analysis

Medium components, myofiber diameter, and qPCR (*MYH*, *SCN*, SR-related, and t-tubule-related genes) were analyzed using an unpaired two-tailed Student’s *t*-test. The expressions of Pax7, MyoD, and Myf5 were analyzed using a *t*-test with Bonferroni correction. The expressions of SCN4A and SCN5A were analyzed using Tukey’s test. Data are presented as means ± SEM. *p* < 0.05 was considered statistically significant.

## Data Availability

The raw data supporting the conclusion of this article will be made available by the authors without undue reservation.

## Supplementary Material

Supplementary Figure S1 Purification of iMuSCs from the mesoderm cell population by flow cytometry using *PAX7*-Venus fluorescence. **(A)** Schematic of the iMuSC differentiation protocol. **(B)** Representative images of Venus-fluorescent-positive iMuSCs. Scale bars, 200 µm. **(C)** Arrow indicates Venus-positive fraction (P5) in the flow cytometry map.

Supplementary Figure S2 Purification of iMuSCs from the mesoderm cell population by flow cytometry using CD57 and CD82 antibodies. **(A)** Schematic of the flow cytometry data. CD57-negative and CD82-positive cells are located in the P5 fraction as purple dots. **(B)** The mRNA expressions of Pax7, MyoD, and Myf5 were quantified by real-time qPCR in every fraction (CD57- CD82+, CD57+ CD82+, CD57- CD82-, CD57+ CD82-) purified by flow cytometry. The expression level of these three genes in unsorted samples was set as the control. The vertical axis of the graph shows the relative expression level normalized by b-actin, the housekeeping gene. The expression level in each fraction was standardized by the expression level of the unsorted (unsort) sample. (The relative expression of Pax7 in the unsorted sample is 1.) Pax7, MyoD, and Myf5 were abundantly expressed in the CD57–CD82+ fraction compared with the unsorted sample. **(C)** The protein expression of Pax7 in every fraction by immunocytochemistry. (Left) Pax7-positive cells were abundant in the CD57–CD82+ fraction compared with those in the other fractions (CD57+ CD82+, CD57- CD82-, CD57+ CD82-). *<0.05 **<0.01, unpaired two-tailed Student’s t-test.

Supplementary Figure S3 Representative images of myotubes on days 5 **(A)**, 7 **(B)**, 14 **(C),** and 42 **(D)** of the culture. Right panels are magnified images. Scale bars, 100 µm (left) and 100 µm (right).

Supplementary Figure S4
**(A)** Schematic of the 3D Matrigel covering. Blue (myotube), beige (differentiation medium; DM), and red (Matrigel mixture). **(B)** Schematic of the two conditions (m-condition and c-condition) for the differentiation. **(C,D)** Representative images of myotubes (anti-MHC, green) in c-condition and m-condition. There was a significant increase in the size of *PAX7*-Venus and CD57-CD82+ iMuSC-derived myotubes at day 42 of the culture (*n*=45 and *n*=46 myotubes from control and Matrigel samples, respectively). Scale bar, 100 µm.

Supplementary Figure S5 The mRNA expression of the myosin heavy chain family (*MYH2, MYH3, MYH4, MYH7, and MYH8*) *,* SR-related genes (*Casq1* and *Atp2a1*) , and t-tubule-related genes (*DHPRα, DHPRβ,* and *DHPRγ*) was quantified by real-time PCR. The expression level of each gene in *PAX7*-Venus iMuSC-derived myotubes on days 14 and 42 between with and without Agrin was shown. Matrigel embedding was performed in both with and without Agrin conditions. The vertical axis of the graph shows the relative expression level normalized by 18S rRNA, the housekeeping gene. The expression level is standardized by the expression level (=1) on day 5. **p* < 0.05, ***p* < 0.01 (unpaired two-tailed Student’s t-test). Error bars indicate the standard error of mean (SEM). In each group (day 14; Agrin+ Agrin-, day 42; Agrin+ Agrin-), three independent samples were used for the analysis.

Supplementary Video S1 Twitching myotubes in m-condition at day 28 of culture.
