# Supplementary material for: Mature Myotubes Generated From Human-Induced Pluripotent Stem Cells Without Forced Gene Expression
Source: Front Cell Dev Biol. 2022 May 30;10:886879. doi: 10.3389/fcell.2022.886879 (PMC9189389; doi:10.3389/fcell.2022.886879)

Supplementary Table 1. The list of primers and antibodies.

(A) Primers

*MYH3* F; gcagattgagctggaaaagg

*MYH3* R; tcagctgctcgatctcttca

*MYH4* F; tgcaatgaagactctggctttc

*MYH4* R; tttttgccacctttctttcca

*MYH8* F; atttccaccaagaaccca

*MYH8* R; aaaggattctgcctctgg

*Casq1* F; atggcgagttttctgctgac

*Casq1* R; ctgcagctctcgttcacctt

*Atp2a1* F; ctgtgtggctgtctggctta

*Atp2a1* R; caggaagaccttcggggatg

*DHPRα* F; aggagcagggagagactgag

*DHPRα* R; aatgtagcacctcagtgggc

*DHPRβ* F; cattgagcgctccaacacac

*DHPRβ* R; atggtgtcagcatccagagc

*DHPRγ* F; gagggactatctgctgcgac

*DHPRγ* R; caatcatgcgcttcaccgac

(B)Antibodies

Anti-MHC(MF20 R&D Systems 1:800)

Anti-MYH1/2 (FAST) (abcam ab51263 1:100)

Anti-MYH4 (FAST) (DSHB BF-F3 1:100)

Anti-MYH2 (FAST) (Millipore MABT840 1:100)

Anti-MYH7(SLOW) (Santacruz sc-53089 1:200)

Anti-MYH3 (embryonic)(Sigma HPA021808 1:200)

Anti-MYH8 (neonatal) (Novus NBP2-41309 1:200)

Supplementary Table 2. The estimated parameters for the intermediate potentials ($V_{1/2}$) and slope factors ($k$).


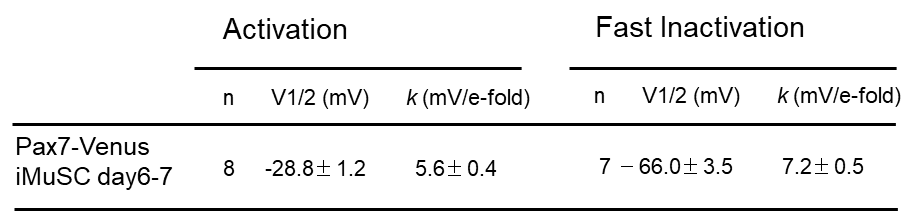

Supplement: Supplementary file 1 [file Table1.DOCX]
