# Supplementary material for: Mature Myotubes Generated From Human-Induced Pluripotent Stem Cells Without Forced Gene Expression
Source: Front Cell Dev Biol. 2022 May 30;10:886879. doi: 10.3389/fcell.2022.886879 (PMC9189389; doi:10.3389/fcell.2022.886879)

Supplementary Figure 1

(A)

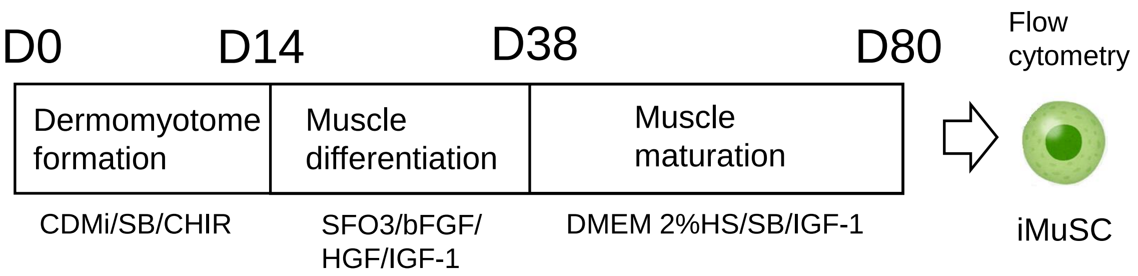

(B)

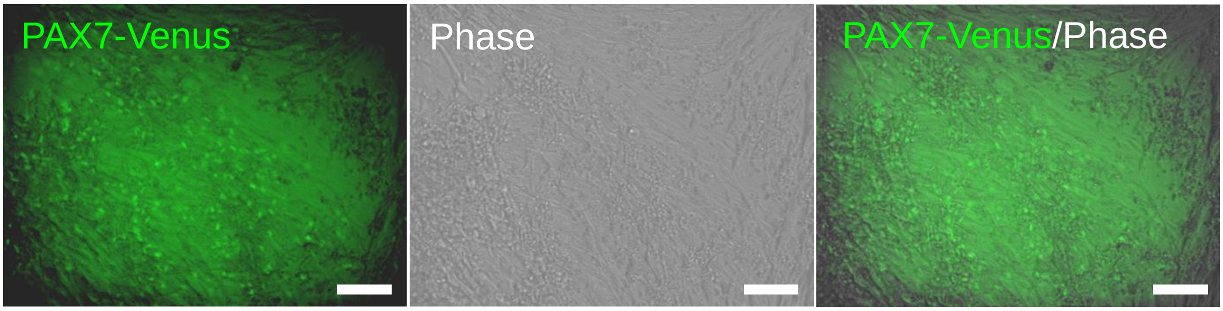

(C)

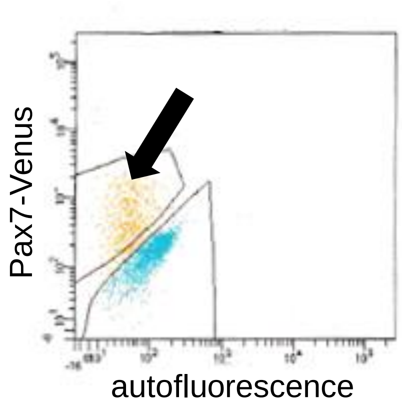

Supplementary Figure 2

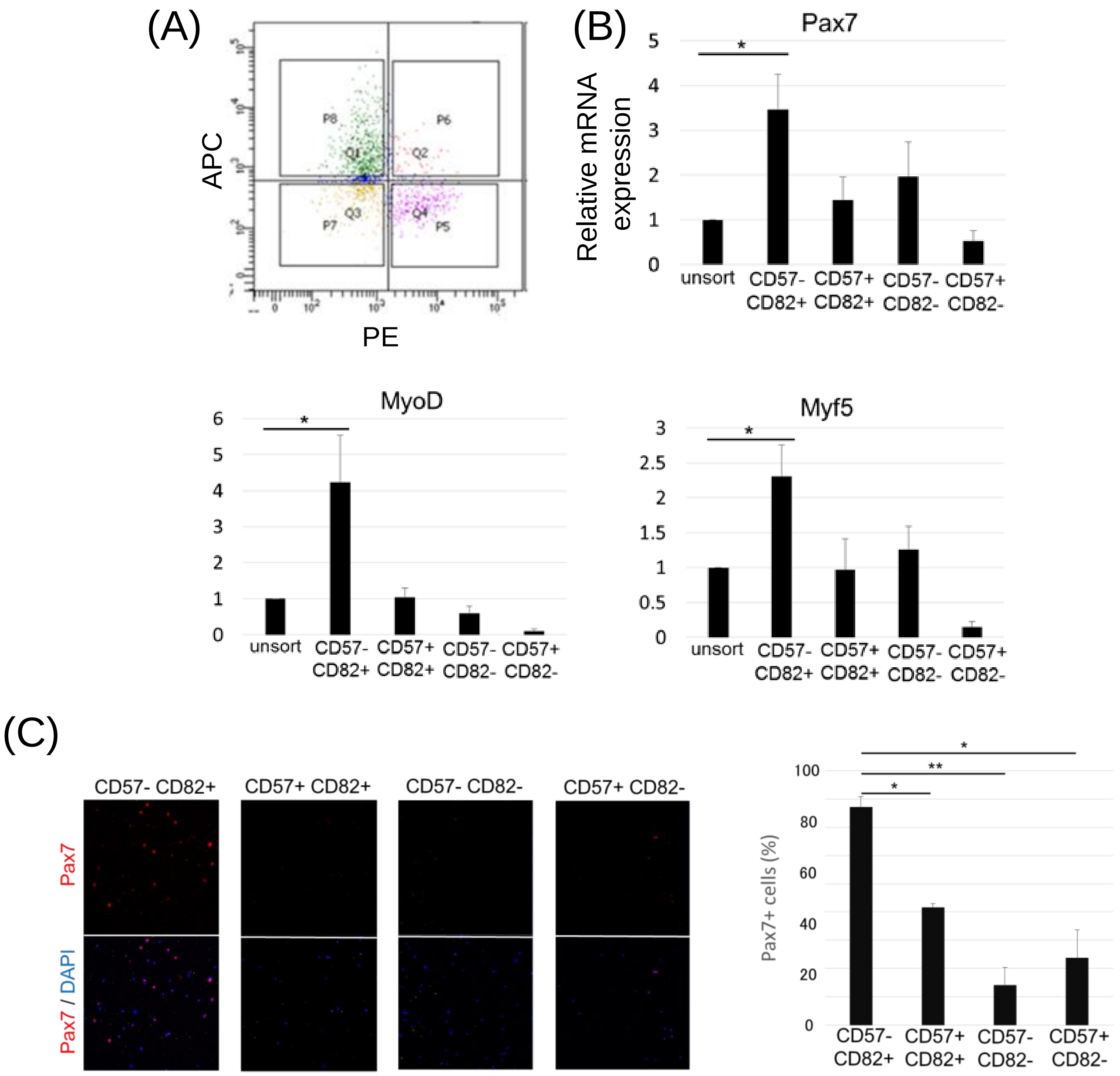

Supplementary Figure 3

(A) Day5

(B) Day7

(C) Day14

(D) Day42

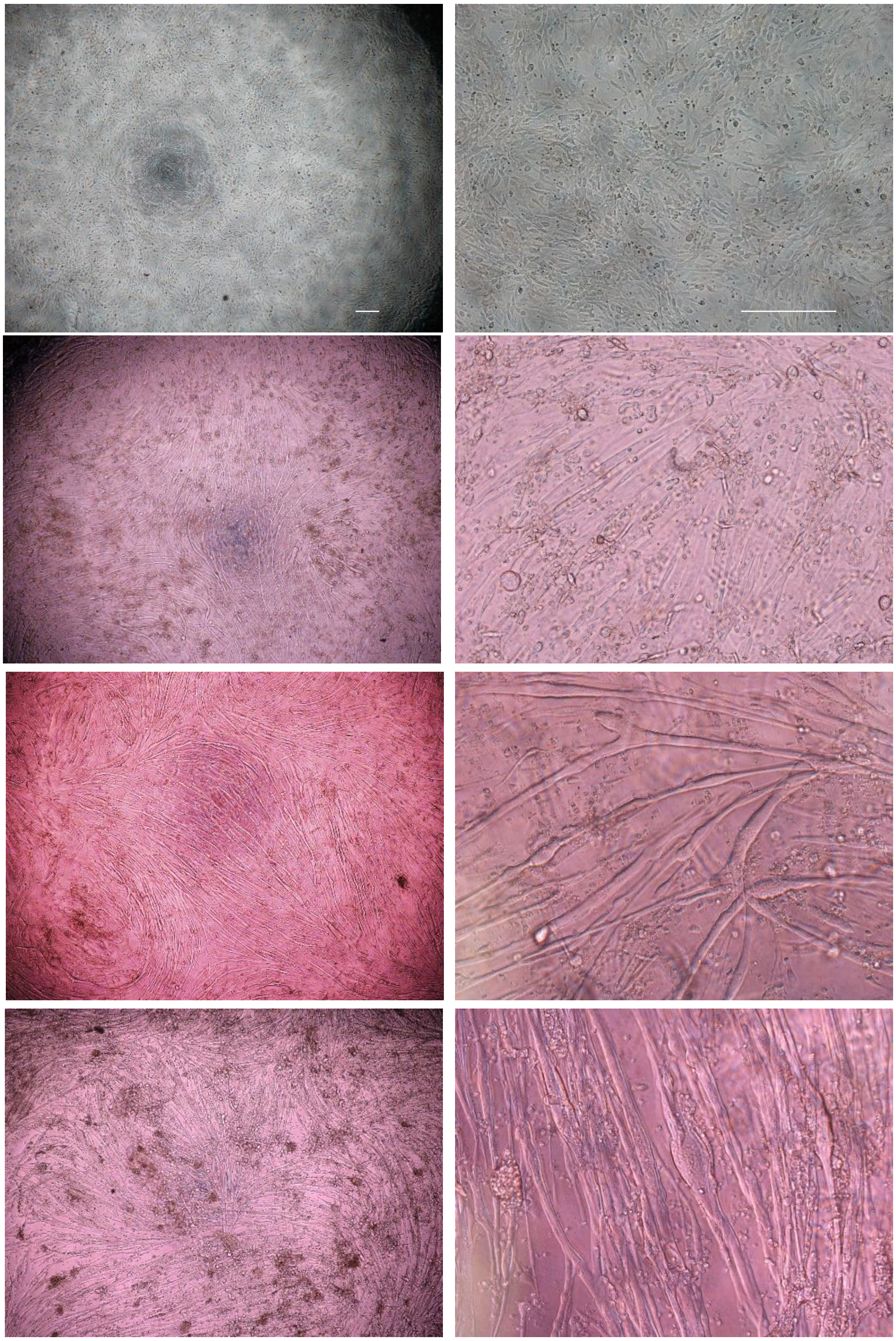

Supplementary Figure 4

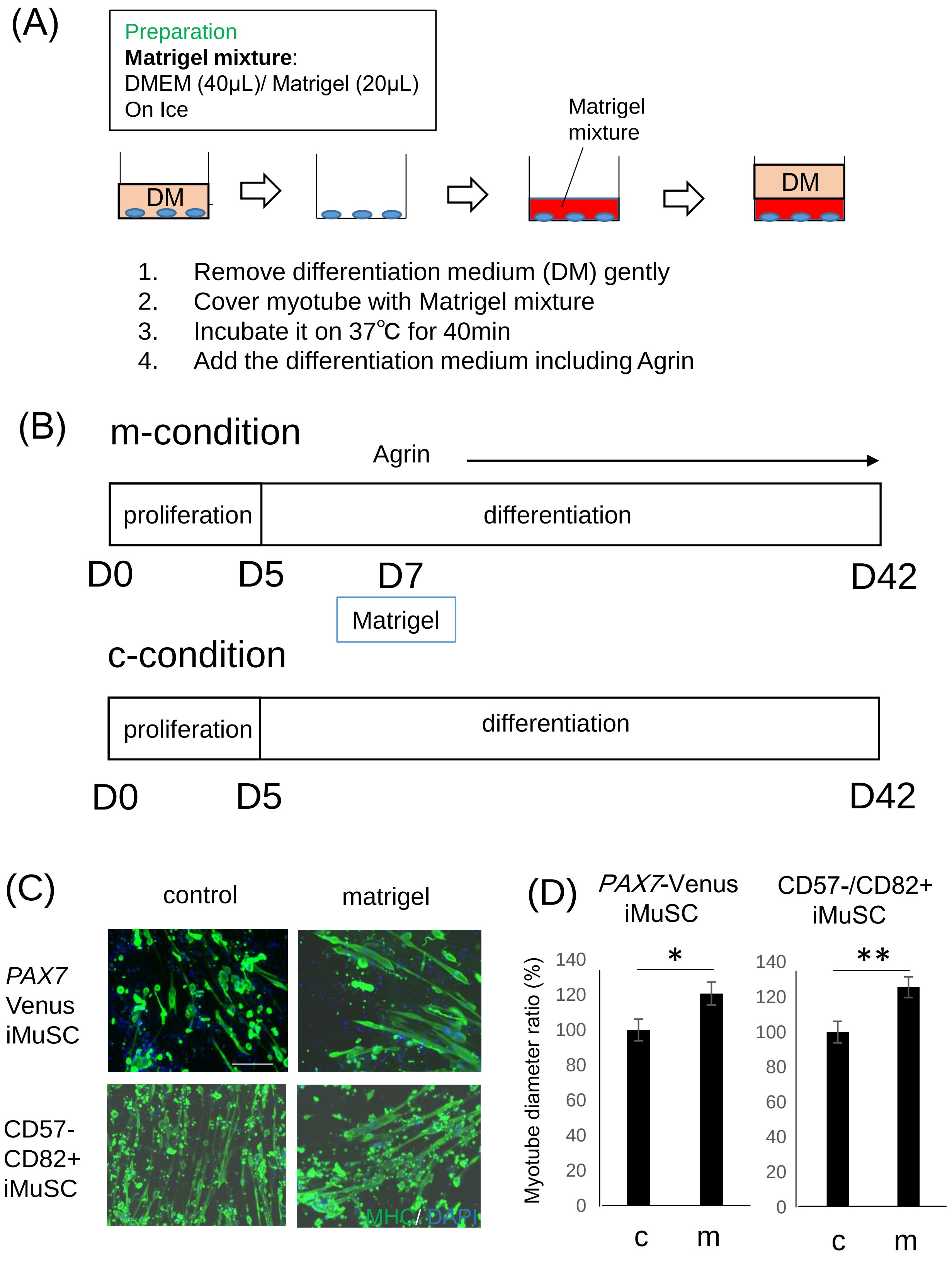

Supplementary Figure 5

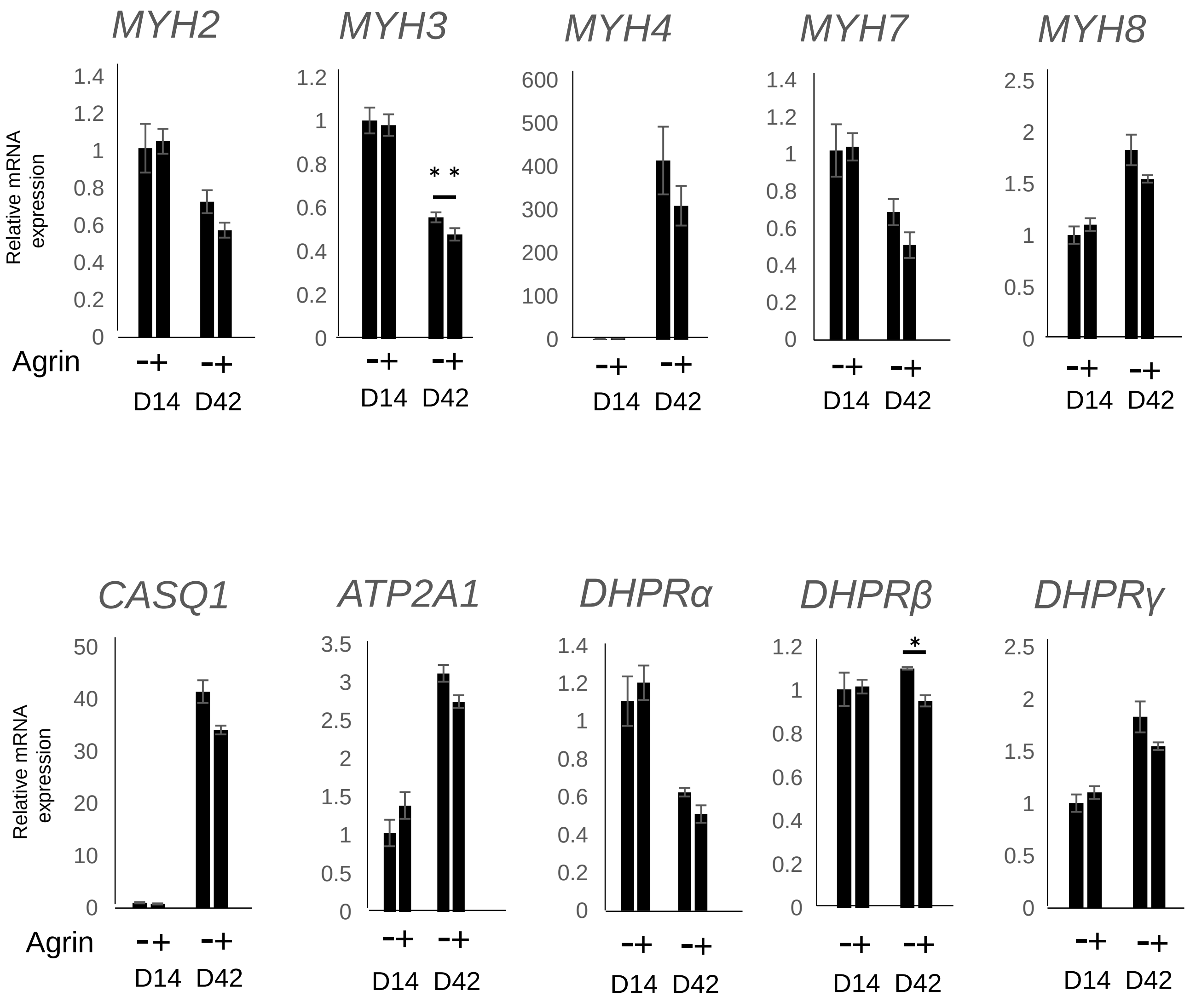

Supplement: Supplementary file 2 [file DataSheet1.pdf]
